# Horizons in Gerontology: A Chat With NIA Senior Leadership

**DOI:** 10.1093/geroni/igaf122.1553

**Published:** 2025-12-31

**Authors:** Kenneth Santora, Kenneth Santora

## Abstract

The National Institute on Aging (NIA) at the National Institutes of Health, Department of Health and Human Services, supports research to understand the nature of aging and to extend the healthy, active years of life. In the decades since its establishment, NIA has propelled advances and innovations across multiple domains of aging research, including basic biology, neuroscience, geriatrics and clinical gerontology, and behavioral and social science. NIA is also the federal leader in Alzheimer’s disease research. This multidisciplinary research portfolio affords a wide array of opportunities in aging science, for early-career and established investigators alike. This symposium will provide a forum for discussion between the research community and NIA leadership, explore research foci of NIA’s scientific divisions, and review opportunities and resources available to the research community.

